# Dysmorphic Uterus: More Questions than Answers

**DOI:** 10.3390/jcm13154333

**Published:** 2024-07-25

**Authors:** Aikaterini Selntigia, Livia Pellegrini, Francesco Gebbia, Daniela Galliano

**Affiliations:** IVIRMA Global Research Alliance, IVIRMA Roma, 00169 Rome, Italy; aikaterini.selntigia@ivirma.com (A.S.); livia.pellegrini@ivirma.com (L.P.); francesco.gebbia@ivirma.com (F.G.)

**Keywords:** dysmorphic uterus, reproductive outcomes, artificial reproductive treatment

## Abstract

A T-shaped uterus is a rare uterine malformation that is classically associated with diethylstilbesterol (DES) exposure. Surprisingly, the prevalence of T- and Y-shaped uterus has increased in recent years despite the absence of a diagnostic consensus and a correlation with the reproductive outcomes has been observed. A systematic electronic database search for all English-language studies published on reproductive outcomes associated with dysmorphic uteri over the past 10 years using PubMed, Google Scholar, and Scopus was performed. This uterine malformation is associated with impaired reproductive outcomes, including primary infertility, miscarriage, ectopic pregnancy, and preterm birth. Hysteroscopic metroplasty is a simple surgical procedure that could potentially improve outcomes in subfertile women, but the data are not robust. Studies reported significant improvements in implantation and pregnancy rates after corrective metroplasty in women undergoing in vitro fertilization. However, multicenter, prospective, randomized, and controlled trials are needed to validate these findings and to help define clear diagnostic criteria, surgical indications, and appropriate follow-up of reproductive outcomes after the procedure.

## 1. Introduction

Female genital tract malformation is a complex topic. Diagnosis is challenging, and the reproductive impact of malformation on spontaneous pregnancy and in vitro fertilization (IVF) and outcomes after metroplasty procedures are not clear. The estimated prevalence of Mullerian anomalies is approximately 5% among women in the general population and up to 13% in women with infertility [1]. Although these uterine malformations are frequently asymptomatic, they can seriously impair obstetric outcomes [1]. In recent years, particular attention has been paid to the dysmorphic uterus, including T-shaped, Y-shaped, and I-shaped uteri. Notably, until the 1980s, a T-shaped uterus was described as a DES-related congenital uterine anomaly, with a cavity shape resembling the letter T based on hysterosalpingography (HSG) imaging. Nevertheless, it is still found in young women during the second and third decades of life, despite DES last being used in 1971 [2]. Among women who were not exposed to DES in utero, a T-shaped uterus may be either congenital or acquired secondary to intrauterine adhesions, adenomyosis, or infectious diseases like tuberculosis [3].

The prevalence of a T-shaped uterus and its impact on reproductive outcomes are unknown. The presence of uterine anomalies is not typically evaluated among women in the general population who are asymptomatic. Therefore, published studies on a T-shaped uterus have been conducted on patients with a history of adverse reproductive outcomes, such as subfertility, miscarriage, preterm birth, or ectopic pregnancy [4]. The prevalence of T-shaped uterus in women with poor reproductive outcomes is up to 0.8% [5]. In these symptomatic women, hysteroscopic metroplasty has been considered a treatment option, but there is no robust evidence regarding its efficacy or safety [5].

This study aims to review available information on the diagnostic criteria, prevalence, clinical relevance, and effectiveness of metroplasty for dysmorphic uteri. In particular, it is important to investigate uterine factors affecting women undergoing IVF with primary infertility, recurrent miscarriage, and repeat implantation failure (RIF). The study will also highlight areas where future research should be focused.

## 2. Materials and Methods

### 2.1. Search Strategy

We conducted a systematic electronic database search for all English-language studies published on reproductive outcomes associated with dysmorphic uteri over the past 10 years (January 2013–March 2024) using PubMed, Google Scholar, and Scopus. The search terms used were (dysmorphic uterus) AND (diagnosis: ultrasound 3D magnetic resonance imaging-hysteroscopy); (dysmorphic uterus) AND (infertility); (dysmorphic uterus) AND (reproductive outcome). The most recent electronic search was conducted in March 2024. We followed the Preferred Reporting Items for Systematic Reviews and Meta-Analyses (PRISMA) guidelines [6].

### 2.2. Selection Process and Data Collection

Titles and abstracts were independently reviewed by three authors (A.S., L.P., and D.G.). After the initial screening of titles and abstracts retrieved by the search, the full texts of all potentially eligible studies were assessed. The primary outcomes of interest were pregnancy rate, spontaneous pregnancy, IVF pregnancy rate, live birth rate (LBR), miscarriage rate, preterm delivery, and ectopic pregnancy. Descriptive tables were used to present data on diagnostic criteria, reproductive outcomes, and the effects of surgical treatment.

Duplicates were removed after the initial selection, and further articles were excluded if their title, abstract, or materials and methods did not align with the objectives of our review. The full texts were examined for eligibility, and articles meeting the inclusion criteria were selected. We included prospective and retrospective cohorts, case-control studies, and observational studies. Literature reviews, case reports, and abstracts from scientific meetings were not included. One reviewer (A.S.) followed specific parameters to collect the data, and a second reviewer (L.P.) independently repeated the process for the entire set of included publications. No discrepancies were found between the two reviewers.

## 3. Results

**Figure 1:**
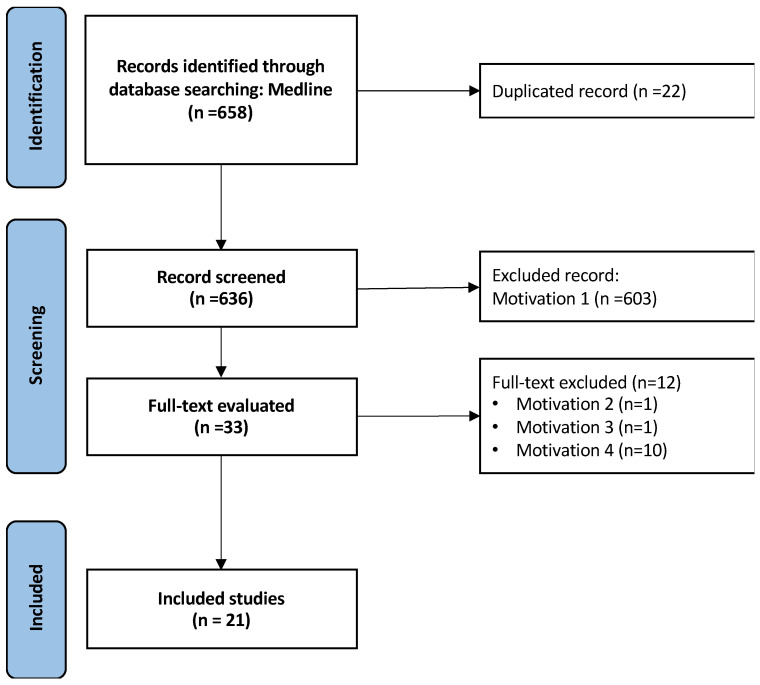
Flow diagram of study identification and selection (PRISMA).

The article selection process is detailed in Figure 1. Initially, 658 records were identified through electronic searches. After exclusions, 21 studies were included in this review.

Motivation 1 = only abstract; motivation 2 = video article; motivation 3 = comment; motivation 4 = review.

### 3.1. Diagnostic Tools

There is no consensus regarding the gold standard method for diagnosing dysmorphic uterus. The best tool for examining the morphology of the uterine cavity is three-dimensional (3D) ultrasound, as neither HSG nor hysteroscopy provides information on uterine wall thickness or serosal contour [7]. Three-dimensional ultrasound appears to be the most appropriate method for diagnosing congenital uterine anomalies owing to its high accuracy, availability, and low cost [5]. Of the studies included in our review, 19 reported diagnostic tools and/or criteria for dysmorphic uterus [3,8,9,10,11,12,13,14,15,16,17,18,19,20,21,22,23,24,25]. The most commonly cited diagnostic method was 3D transvaginal ultrasound (TVS) (11 studies). It was the only diagnostic tool used in seven of these studies [5,6,7,9,15,26,27] and the diagnosis was confirmed by hysteroscopy in the remaining four studies [12,13,14,16]. Seven studies mentioned HSG as a diagnostic tool; five of these used only HSG [11,16,17,19,20]; and two used HSG confirmed by hysteroscopy [9,16]. Four studies mentioned hysteroscopy alone [16,18,21,28]. Only one study mentioned magnetic resonance imaging (MRI) [20]. Notably, 3D ultrasound has a high sensitivity and specificity for congenital uterine malformations (100% and 99%, respectively) [29].

Table 1 describes the complexity of current ultrasonographic classifications, and highlights the need for a classification that ensures diagnostic accuracy. According to the historical American Fertility Society classification from 1988, a T-shaped uterus is a congenital uterine DES drug-related malformation [22]. More recently, the European Society of Human Reproduction and Embryology and the European Society for Gynaecological Endoscopy (ESHRE/ESGE) classification system for female genital tract anomalies recognized the clinical significance of T-shaped uterus and uterus infantilis, categorizing them as Class 1 uterine anomalies (U1), also known as “dysmorphic uteri” [23]. The T-shaped uterus (Class U1a) is characterized by a narrow uterine cavity due to thickened lateral walls with a correlation of 2/3 uterine corpus and 1/3 cervix. A T-shaped uterus should be differentiated from uterus infantilis, which is also characterized by a narrow uterine cavity, but is without lateral wall thickening and has an opposite correlation of 1/3 of the uterine body and 2/3 of the cervix [23]. In 2022, a proposal for a modification to the ESHRE/ESGE classification of dysmorphic uterus was suggested with the addition of a “Y-shaped uterus” under U1b, described as “a uterus characterized by a narrow uterine cavity due to thickened lateral walls and a fundal indentation of <50% of the uterine wall thickness at the midline level, with a correlation 2/3 uterine corpus and 1/3 cervix” [24]. There were no specified objective criteria to diagnose a T-shaped uterus or explain how the uterine measurements should be performed.

In 2020 a new classification system was proposed by the Congenital Uterine Malformation by Experts (CUME) to describe ultrasound features of a T-shaped uterus [3]. CUME was the first US classification to use three objective morphometric measurements gathered from coronal view images using 3D ultrasound to describe uterine morphology in clinical practice. This included normal/arcuate uterus, borderline T-shaped, and T-shaped uterus with specific cut-off levels: lateral indentation angle ≤ 130°; lateral internal indentation depth ≥7 mm; and T-angle ≤ 40°. The presence of two of these three criteria is defined as a borderline T-shaped uterus, whereas a lack of these criteria or the presence of only one is defined as a normal uterus with respect to lateral uterine morphology. This definition was supported by testing the reliability and diagnostic accuracy of the measurements and by a post-test probability of >90% in the target population. The diagnosis made most often by experts was used as the reference [3].

A recent study was the first to evaluate the degree of concordance between three objective morphometric measurements from coronal 3D ultrasound images for the diagnosis of a Y-shaped uterus. A 100% inter-observer agreement (two separate physicians) was achieved using the following measurements: lateral indentation depth 4–7 mm and fundal indentation depth 5–9 mm; lateral indentation angle 121–149° and fundal indentation angle 121–145°; and Y-angle 25–46° [15].

In 2021 Alonso Pacheco et al. proposed the “Rule of 10”. Accordingly, a uterus is considered T-shaped if the length of the intracavitary line drawn parallel to the interostial line at 10 mm from it is ≤10 mm (R10 ≤ 10 mm) [25]. In contrast, Salim et al. and the 2020 American Society for Reproductive Medicine classification did not include a T-shaped uterus as a malformation [30].

These US classifications highlight the absence of clear definitions and objective diagnostic criteria, making it difficult for clinicians to accurately diagnose a dysmorphic uterus and to perform a differential diagnosis.

Kougioumtsidou et al. evaluated the concordance between 3D-TVS and hysteroscopy with laparoscopy for determining the type and classification of uterine anomalies. They found concordance in all those with septate uterus, dysmorphic uterus, bicorporeal, hemi-uterus or unicorporeal, and aplastic uterus, as well as in one of two with a normal uterus. The sensitivity, specificity, positive predictive value, and negative predictive value for dysmorphic uterus were all 100% (kappa index [k] = 1.00). Overall, 3D-TVS showed perfect diagnostic accuracy (k = 0.945) in the detection of congenital uterine anomalies [31].

There is a high degree of concordance between MRI and 3D-TVS (kappa index, 0.880; 95% CI, 0.769–0.993) in the diagnosis of uterine malformations, and the relationship between cavity and fundus is equally well-visualized with both techniques [32]. Magnetic resonance-HSG is a well-tolerated technique demonstrating high accuracy (sensitivity 91.7%; 95% CI 61.5–99.8 and specificity 92.9%; 95% CI 66.1–99.8) for investigating tubal patency and intra-uterine abnormalities during a diagnostic work-up of female infertility.

It has been proposed dysmorphic uterus could be differentiated into more specific subtypes using 3D-TVS and hysteroscopy. In addition to the narrowing of the middle third of the cavity caused by the thickened lateral walls, the Y-shaped uterus has thickening at the fundus leading to a small indentation into the uterine cavity. On hysteroscopy, the uterine cavity has a tubular shape with an indentation at the fundus. This indentation gives the uterus the Y-shape. It displaces the tubal ostia distally and laterally, which reduces the interstitium distance when compared with the T-shaped uterus and I-shaped uterus, in which lateral wall thickening extends from the internal cervical to the fundus. There is almost no difference between the upper, middle, and lower parts of the uterine cavity. On hysteroscopy, the entire uterine cavity has a tubular shape up to the fundus with the interostial distance significantly reduced. Both tubal ostia were easily visualized because of their proximity [33].

**Figure 2:**
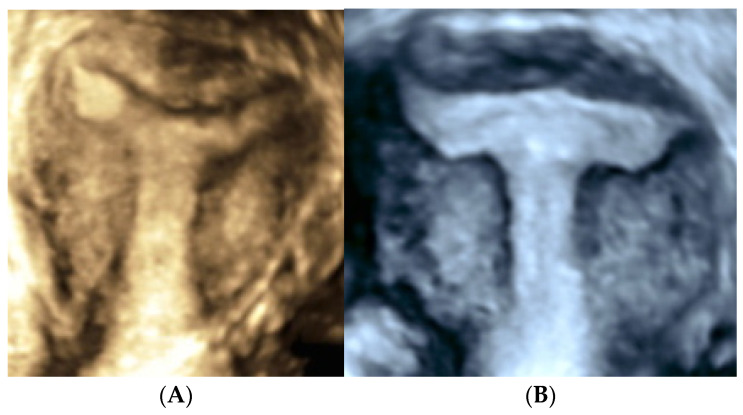
(**A**) Transvaginal three-dimensional ultrasound images of a Y-shaped uterus. (**B**) Transvaginal three-dimensional ultrasound images of a T-shaped uterus.

All of the previous ultrasound classifications and the different tools used to diagnose and evaluate dysmorphic uteri highlight the absence of substantial objective and reliable evidence that can be replicated for the definition and classification. Objective diagnosis is key to avoiding overdiagnosing conditions that will never cause harm, as well as underdiagnosing harmful and potentially curable conditions. Figure 2 shows two images of a T-shaped and Y-shaped uterus.

### 3.2. Hysteroscopic Metroplasty and Reproductive Outcomes

Dysmorphic uteri are associated with impaired reproductive outcomes such as primary infertility, miscarriage, repeat implantation failure, and preterm birth. Hysteroscopic metroplasty is a simple procedure that could potentially improve outcomes in subfertile women, but the data are not robust. Reproductive outcomes of women with symptomatic uterine malformations after surgical correction with metroplasty were reported in 13 of the 20 selected full-text articles.

Hysteroscopic metroplasty is a minimally invasive procedure that involves inserting a hysteroscope through the vagina and cervix into the uterus, enabling direct visualization of the uterine cavity. Surgical correction is then performed using specialized instruments inserted through the hysteroscope. However, a dysmorphic uterine cavity comprises different structural anomalies that require different metroplasty techniques. The choice of surgical approach depends on the type and severity of the uterine irregularity, the patient’s reproductive objectives, and the surgeon’s expertise. In complex cases, a combination of different surgical approaches may be employed. Table 2 shows the different surgical methods used in the studies included in this review.

Three studies included in our analysis examined reproductive outcomes in women with dysmorphic uteri who did not undergo metroplasty surgical intervention, despite the diagnosis. One study reported a non-significant difference in LBR in women with T-shaped uteri compared with normal uteri (66.5% vs. 40%, *p* = 0.19) [34]. This study reported a statistically significant increase in the risk of clinical miscarriage (33%) and ectopic pregnancy (8.9%) in women with T-shaped uterus compared with normal controls. Marianna et al. reported that the rates of biochemical pregnancy in normal and T-shaped uteri were 58.6% and 51.9%, respectively, but the difference was not statistically significant. However, women with dysmorphic uterus had higher rates of preterm deliveries, miscarriages, and ectopic pregnancies, and there was a trend towards a lower LBR. This trend did not achieve statistical significance (40.0% versus 66.5% for normal uteri and 65.7% for intermediate cavity morphology; *p* = 0.19) [27].

The main aim of this review was to understand reproductive outcomes after metroplasty procedures in women with dysmorphic uteri, as outlined in Table 2. These outcomes can vary depending on factors such as the type and severity of the uterine anomaly, the specific metroplasty procedure employed, and individual baseline characteristics.

Using metroplasty to correct structural abnormalities such as a T-shaped or Y-shaped uterus in symptomatic patients can improve reproductive outcomes. Analysis of the 13 full-text papers included in this review showed overall improvements in spontaneous and IVF pregnancy rates regardless of the surgical metroplasty procedure employed, type of infertility (e.g., primary infertility or recurrent miscarriage), or type of uterine morphology (T-shaped or Y-shaped). All studies reported improved reproductive outcomes in patients with dysmorphic uterus following metroplasty. Pregnancy rates ranged from 36% to 78% [10,11,12,13,14,15,16,17,18,19,27,34] and LBRs ranged from 52% to 82%. Interestingly, an improvement was also seen in spontaneous pregnancy during the first 6 months to 1 year of follow-up after metroplasty, with nine studies reporting spontaneous pregnancy rates ranging from 3% to 56% [1,9,11,14,15,16,18,19,27,34]

By optimizing uterine cavity morphology, metroplasty can improve embryo implantation, leading to higher pregnancy rates and LBRs. Ten studies reported pregnancy rates after IVF procedures and an important improvement ranging from 21% to 60% was observed [9,10,11,12,14,15,16,18,19,27,34]. Notably, four studies included patients with RIF whose LBR rose by up to 77% after metroplasty [9,11,16,34]. One study reported LBRs of 66.5% in women with normal uteri and 40.0% in women with T-shaped cavity morphology [35]. Following successful metroplasty, therefore, there is often an increase in LBRs among individuals with uterine malformations.

Metroplasty can also significantly reduce the risk of future miscarriages in women with uterine anomalies. Surgical correction of these anomalies can support normal embryo development and implantation, increasing the rate of healthy pregnancies and reducing the likelihood of pregnancy loss. Miscarriage rates after metroplasty ranged from 7.9% [18] to 22.0% [10,11,15]. For women with primary infertility, miscarriage rates after metroplasty ranged from 9% [11] to 25.0% [12]. Among women who had experienced recurrent early miscarriages, rates after metroplasty ranged from 12.2% [21] to 40.0% [13].

Dilbaz et al., 2022, compared the reproductive outcomes after metroplasty in women with T-shaped and Y-shaped uteri and reported a marked improvement in the group with Y-shaped uteri in terms of LBR, with rates of 36% and 53%, respectively [14].

Only one study compared IVF outcomes with and without metroplasty in women who presented with RIF and a T-shaped uterus. Interestingly, this study observed statistically significant improvements after metroplasty in pregnancy rates (56% vs. 26% without surgery) and clinical pregnancy (42% vs. 17% without surgery). In contrast, the difference in miscarriage rates was not significant [8].

While metroplasty addresses uterine structural issues and improves obstetric outcomes, it does not appear to affect ectopic pregnancy risk. Ectopic pregnancy rates ranged from 0% to 8% depending on the study and the type of uterine anomaly [14,17,27].

Only one study reported intraoperative complications, with a rate of 2% based on one cervical laceration and one case of false passage [15]. No other studies reported complications. Although metroplasty aims to improve fertility and reduce miscarriage risk, obstetric complications such as preterm delivery, placental abnormalities, and cesarean section may still occur. The preterm delivery rate in particular remained high following surgical corrections, with rates ranging from 4.7% [16] to 35.0% [13]. Three studies reported placenta accreta spectrum diseases after metroplasty, with rates ranging from 6% to 21% [10,12,18]. The study which better analyzed the data of abnormal placentation demonstrated that of eight women who had a retention of deciduous tissue after delivery, seven were treated conservatively and one underwent a hysterectomy for placental accreta. Although this may suggest a possible correlation between metroplasty and the risk of placental remnants, the small sample size of this study precludes us from drawing a firm conclusion. Moreover, it should be stressed that the only woman who required a hysterectomy due to placental accreta had a personal history of repeated uterine curettages after spontaneous abortions [12].

The likelihood of cesarean section or vaginal birth after metroplasty depends on various factors, including the type of uterine abnormality corrected, individual health considerations, and the obstetrician’s recommendations. Our results showed that the probability of undergoing a cesarean section ranged from 38.9% [21,22] to 88.2% [8]. Currently, data are heterogeneous and no recommendations are available on the delivery route for women with a previous surgery for dysmorphic uterus, so management decisions should be made in consultation with healthcare providers while considering the potential risks and benefits.

## 4. Discussion

It is currently challenging to manage patients with primary infertility, RIF, or recurrent miscarriage and dysmorphic uterus. In the relevant studies included in this review, the prevalence of a T-shaped uterus was up to 1.5% among women with poor reproductive outcomes [20,36].

The etiology of a T-shaped uterus remains unknown, although different studies considered it to be of the primary origin or secondary to adhesions or adenomyosis. Moreover, there is no universal definition for a T-shaped or Y-shaped uterus, and diagnosis is performed using different methods (mainly 3D ultrasound and hysteroscopy as mentioned above). Accordingly, all studies included in this review used different inclusion criteria for women with a history of impaired reproductive outcomes as well as different diagnostic methods and different criteria for classifying T-shaped and Y-shaped uteri. Additionally, the surgical technique used for treating the condition varied between the studies. All these factors highlight the heterogeneity in clinical management and the need for consensus in the use of diagnostic tools and the selection of treatment.

One of the key limitations is the absence of a consensus on diagnostic criteria for T-shaped and Y-shaped uterus. In this review, most of the studies used 3D-TVS or hysteroscopy. For 3D-TVS diagnosis, there are six different ultrasonographic classifications and most use subjective criteria for diagnosis (Table 1), except for the CUME classification, which includes fairly strict objective criteria. In this review, eight studies used the ESHRE/ESGE definition for T-shaped and Y-shaped uterus [10,12,13,14,15,20,26], while only three studies used CUME classification criteria [5,27].

Pacheco et al. [37] have published diagnostic criteria based on hysteroscopy, but the main issue remains the indication for a hysteroscopic exam. According to the literature, hysteroscopy is not effective in increasing IVF success in women with primary infertility without RIF [38]. However, it is important to highlight the impact of uterine factors on IVF success. When office-based hysteroscopy was performed in women undergoing their first assisted reproductive treatment (ART), the incidence of uterine abnormalities ranged between 11% and 22%, which affected overall outcomes if the abnormality was not corrected [39]. When the study population consisted of women with RIF, the incidence of uterine defects increased from 26% to 45% [39]. The most reported uterine abnormalities were endometrial polyps, small fibroids, septa, and adhesions.

Nevertheless, considering the degree of concordance between 3D-TVS plus HSG and hysteroscopy (k = 0.77; 95% CI 0.6–0.84) and the shorter and more patient-friendly 3D-TVS procedure relative to hysteroscopy, 3D-TVS should be employed in the diagnosis of uterine malformations. Despite limited evidence demonstrating that uterine abnormalities may impair IVF success, we recommend a careful study of the uterus before starting an ART cycle. This integrated approach makes a distinction between women undergoing ART for the first time, and women suspected to have RIF, even in the presence of a morphologically normal uterus [39].

It is challenging to investigate the prevalence and effects of dysmorphic uteri in the general population since the studies conducted until now have included symptomatic patients with poor reproductive outcomes. Most studies evaluated reproductive outcomes after metroplasty for dysmorphic uterus in symptomatic patients and agreed that there were improvements in pregnancy rates, LBRs, and miscarriage rates [7,9,10,11,12,14,15,16,17,18,19,27,34].

Only three studies compared reproductive outcomes in women with normal uteri and women with dysmorphic uteri who did not receive metroplasty. These studies demonstrated a non-significant difference in pregnancy rates between these groups, but a significantly higher miscarriage rate in the dysmorphic group [8,26,36].

Only one study investigated IVF outcomes in women with RIF and T-shaped uteri by comparing those who did and did not receive hysteroscopic metroplasty surgery. Interestingly, this study reported a statistically significant improvement in pregnancy rates in women who received surgery (56% vs. 26% without surgery) and clinical pregnancy after metroplasty (42% vs. 17% without surgery). The difference in miscarriage rates was not significant [16].

Studies on T-shaped uteri largely included women with impaired reproductive outcomes; surgical treatment was found to be beneficial in all these studies. Moreover, the patients were divided into subgroups (primary infertility, recurrent miscarriage, and RIF) to assess the effect of metroplasty more accurately.

Among women with primary infertility, all studies reported improved reproductive outcomes following metroplasty in women with dysmorphic uterus. Pregnancy rates ranged from 36% to 72% [11,12,13,17,18,27] and LBRs ranged from 53% to 82% [9,11,12,13,17,18]. Notably, spontaneous pregnancy rates also improved during the first 6 months to 1 year of follow-up after metroplasty, with five studies reporting spontaneous pregnancy rates ranging from 19% to 45% [9,11,12,18,27].

Metroplasty can correct dysmorphic uterus and lead to improvements in embryo implantation, higher pregnancy rates, and higher LBRs. Five studies reported considerable improvements in pregnancy rates after IVF procedures in women with primary infertility who received metroplasty, with rates ranging from 20− to 55% [9,11,12,18,27]. The miscarriage rate after metroplasty ranged from 9% to 25% in these women [11,12,18,27].

Among women who had experienced recurrent miscarriages, an overall improvement was observed after metroplasty. The pregnancy rate varied from 71% to 85% because the main issue for these women is not the implantation, but the progression of the pregnancy [11,12,13,18]. Interestingly, the LBR ranged from 60% to 78% [9,11,12,13,18], while the miscarriage rate ranged from 12% to 40% [11,12,13,18].

Women with RIF are another important group in the field of reproductive medicine. Four studies included these women and the results were surprising, with LBRs rising to 77% after metroplasty and a pregnancy rate between 64% and 80% [9,11,16,34].

Only one study compared pregnancy outcomes of patients who received surgery for a T- or Y-shaped uterus. A retrospective study by Dilbaz et al. reported an overall LBR of 76% (38/50) after hysteroscopic surgery. When the T-shaped and Y-shaped groups were compared, the pregnancy rates of the women with recurrent pregnancy loss in the T-shaped uteri group were better than in the Y-shaped uteri group, while similar pregnancy rates were found among women with primary infertility in the two groups. Recurrent pregnancy loss was lower and full-term pregnancy rates were higher in the T-shaped group versus the Y-shaped group [14].

An overall improvement in reproductive outcomes was observed after metroplasty. Considering the results of this review regarding each reproductive outcome and the various subgroups of symptomatic patients, comparable percentages were observed in terms of pregnancy rates, LBRs, and miscarriage rates. However, this review identified important limitations in the recent literature. Firstly, there is an absence of a consensus on diagnostic tools and criteria for T-shaped and Y-shaped uterus. As a result, there is heterogeneity in the metroplasty indication, and the method used. Secondly, there are currently no randomized controlled trials (RCTs) on this topic and the available observational studies use inconsistent diagnostic methods and definitions.

## 5. Conclusions

The prevalence of T-shaped and Y-shaped uteri and the associated clinical reproductive outcomes remain unclear because the available studies use different definitions and diagnostic criteria. Additionally, robust evidence is lacking on the effect of metroplasty on the dysmorphic uterus. Although of low quality, all studies conducted on symptomatic women with dysmorphic uterus agreed that reproductive outcomes improved after metroplasty, whether the pregnancy was spontaneous or followed by IVF treatment. In particular, women who underwent IVF had significant improvements in implantation and pregnancy rates after corrective metroplasty. 3D ultrasound is crucial after metroplasty to evaluate better the uterine morphology before performing an embryo transfer. One of the most challenging issues in reproductive medicine is the management of women with RIF. Although pregnancy rates and LBRs improved after metroplasty in patients with RIF, this review highlights the lack of high-quality evidence on this important topic.

## 6. Future Directions

Future studies should address the need for reliable and universally accepted criteria for diagnosing T-shaped and Y-shaped uteri. In addition, given the limitations of the studies reviewed here, there is a need for multicentre, prospective RCTs to validate these findings.

In the field of reproductive medicine, it is important to assess the real impact of dysmorphic versus normal uteri on reproductive outcomes following spontaneous pregnancy or IVF treatment. If evidence of impaired reproductive outcomes is found, RCTs evaluating the effectiveness and safety of metroplasty are needed before incorporating this intervention into clinical practice.

## Figures and Tables

**Table 1:** All the current classifications regarding the diagnosis of T-shaped uterus, borderline T-shaped uterus, and Y-shaped uterus.

Current Classifications	T-Shaped	Borderline T-Shaped	Y-Shaped
ASRM, 1988	Class VII-DES * related. Letter T based on hysterosalpingography imaging	NA *	NA
CUME, 2020	All of three: - Lateral Indentation Angle (AI) ≤ 130°; - T-angle (AT) ≤ 40°; - Lateral Bulging (LB) ≥ 7 mm	At least two of three	NA
ESHRE/ESGE, 2013	Class U1a narrow uterine cavity due to thickened lateral walls with a correlation 2/3 uterine corpus and 1/3 cervix.	NA	NA
ESHRE/ESGE modified, 2022	Class U1a narrow uterine cavity due to thickened lateral walls with a correlation 2/3 uterine corpus and 1/3 cervix.	NA	U1b narrow uterine cavity due to thickened lateral walls and a fundal indentation of <50% of the uterine wall thickness at the midline level, with a correlation 2/3 uterine corpus and 1/3 cervix
Pacheco, 2021- Rule of 10	R10 ≤ 10 mm	NA	NA
Aslan, 2024	All of three: - Lateral indentation angle (AI) ≤ 130°; - T-angle (AT) ≤ 40°; - Lateral bulging (LB) ≥ 7 mm	NA	All of three: - Lateral indentation depth 4–7 mm and fundal indentation depth 5–9 mm; - Lateral indentation angle 121–149° and fundal indentation angle 121–145°; - Y-angle 25–46°

* DES: diethylstilbestrol, NA: not applicated.

**Table 2:** Summary of reproductive outcomes after metroplasty.

Study	Population Groups	Study Design	N	Diagnosis	Metroplasty	PR *	SPR *	IVF PR *	LBR *	MR *	PD *	Ectopic Pregnancy	Placentation Problems	Complications	CS *
Acet et al., 2022 [9]		prospective	182	HSG + HSC *	Monopolar and bipolar hook needle					NR *	NR	NR	NR	none	NR
	Primary infertility		74				32% (24/74)	51% (28/55)	54% (40/74)	NR	NR	NR	NR	none	NR
	Reccurent miscarriage		60				37% (22/60)	71% (30/42)	76% (46/60)	NR	NR	NR	NR	none	NR
	Repeated implantation failure		48				0	40% (19/48)	40% (19/48)	NR	NR	NR	NR	none	NR
Bilgory et al., 2021 [10]		retrospective	15	3D TVS *	Diode laser	60% (9/15)	NR	60% (9/15)	78% (7/9)	22% (2/9)	29% (2/7)	0	1 placenta previa	none	67% (4/6)
Boza et al., 2018 [11]		prospective	56	HSG+ 3D-TVS	Bipolar hook needle	66% (37/56)	30% (17/56)	36% (20/56)	52% (29/56)	22% (8/37)	10% (3/29)	0	0	none	NR
	Primary infertility		32			63% (20/32)	28% (9/32)	34% (11/32)	53% (17/32)	9% (3/32)	NR	NR	NR		NR
	Reccurent miscarriage		10			80% (8/10)	40% (4/10)	40% (4/10)	60% (6/10)	20% (2/10)	NR	NR	NR		NR
	Repeated implantation failure		14			64% (9/14)	28% (4/14)	36% (5/14)	43% (6/14)	21% (3/14)	NR	NR	NR		NR
Di Spiezio Sardo et al., 2020 [12]		retrospective multicenter	214	3D-TVS +HSC	Bipolar electrode or scissors	73% (156/214)	47% (74/156)	53% (82/156)	80% (125/156)	19% (31/156)	14% (17/125)	0	6% (8/125)	none	54% (68/125)
	Primary infertility		166			72% (119/166)	45% (53/119)	55% (66/119)	81% (96/119)	19% (23/119)	10% (9/96)	0		none	55% (52/96)
	Reccurent miscarriage		48			77% (37/48)	57% (21/37)	43% (16/37)	78% (29/37)	22% (8/37)	28% (8/29)	0		none	55% (16/29)
Di Spiezio, 2015 [13]		prospective	30	3D-TVS +HSC	HOME-DU technique	57% (17/30)			71% (12/17)		35% (6/17)				58% (7/12)
	Primary infertility		22			55% (12/2)			75% (9/12)	25% (3/12)	25% (3/12)	NR	NR	none	
	Recurrent miscarriage		9			71% (5/7)			60% (3/5)	40% (2/5)	60% (3/5)	NR	NR	none	
	Preterm birth		1				0% (0/1)	0	0	0	0	0	0	0	0
Dilbaz et al., 2022 [14]		retrospective	92	3D-TVS +HSC	Monopolar hook needle	54% (50/92)	56% (28/50)	44% (22/50)	76% (38/50)	16% (8/50)	18% (9/50)	8% (4/50)	0	none	NR
	T-shaped		30			37% (11/30)	37% (11/30)	36% (8/22)	53% (16/30)	10% (3/30)	13% (2/30)	0%	0	none	NR
	Y-shaped		62			50% (31/62)	55% (17/62)	64% (14/22)	36% (22/62)	8% (5/62)	32% (7/62)	7% (4/62)	0	none	NR
Ducellier-Azzola et al., 2018 [16]		retrospective	112	HSG or 3D TVS or HSC	Monopolar and bipolar hook needle	62% (100/161)	49% (24/49)	49% (24/49)	60% (60/100)	22% (22/100)	20% (12/60)	NR	NR	2% (2/112)	NR
Haydardedeoğlu et al., 2018 [17]		retrospective	272												
	Primary infertility		162			46% (74/162)	19% (30/162)	20% (33/162)		14% (10/74)	3% (2/74)	1% (1/74)			
	Secindary infertility		110			55% (61/110)	34% (37/110)	22% (24/110)		10% (6/61)	0	2% (1/61)			
J. Ferro [18]	RIF ≥ 5 preembryos transferred	retrospective	190	HSC	Scissors and bipolar hook needle	80% (152/190)	3% (6/190)	77% (146/190)	77% (147/190)	11% (17/190)	22% (34/152)			none	57% (86)
Mutlu, 2022 [19]	RIF ≥ 3 preembryos transferred	retrospective	90	HSG	Monopolar hook needle						5% (4/85)	0		none	88% (15/17)
	T-shaped and metroplasty		48			42% (18/43)	10% (5/48)		30% (13/43)	12% (5/43)					
	T-shaped without metroplasty		42			17% (7/42)				7% (3/42)					
Şükür, et al., 2018 [20]	Infertility	retrospective	97	HSG or MRI	Monopolar needle electrode										
	T-shaped (U1a)		43			37% (16/43)			76% (13/16)	18% (3/43)	12% (2/43)	3%			
	Septate uetrus (U2b)		54			35% (19/54)			84% (16/19)	21% (4/54)	11% (2/54)	0%			
Sánchez-Santiuste, 2020 [21]		prospective	63	HSC	HOME-DU technique	76% (48/63)	36% (13/36)	33% (12/36)	64% (36/63)	8% (5/63)	28% (5/18)	NR	21% (3/36)	none	39% (14/36)
	Infertility		30			67% (20/30)	33% (6/18)	22% (4/18)	62% (18/29)	3% (1/30)	11% (2/18)	NR	NR	none	33% (6/18)
	Recurrent miscarriage		33			85% (28/33)	39% (7/18)	44% (8/18)	67% (18/27)	12% (4/33)	17% (3/18)	NR	NR	none	44% (8/18)
Zhang et al., 2022 [8]		retrospective	111	HSC	Microscissors									none	
	Congenital T-shaped uterus		46			87% (40/46)	33% (15/45)	67% (30/45)	78% (35/44)	20% (9/44)	NR	NR	NR	none	63% (22/35)
	Acquired T-shaped uterus		65			72% (47/65)	56% (29/52)	44% (23/52)	67% (35/47)	23% (12/47)	NR	NR	NR	none	60% (21/35)

* HSG: hysterosalpingography, HSC: hysteroscopy, TVS: transvaginal ultrasound, MRI: magnetic resonance imaging, PR: pregnancy rate, SPR: spontaneous pregnancy rate, LBR: live birth rate, MR: miscarriage rate, PD: preterm delivery, CS: cesarean section, RIF: repeat implantation failure. NR: not reported.
